# Bactericidal Activity of Ready-To-Use Alcohol-Based Commercial Wipes According to EN 16615 Carrier Standard

**DOI:** 10.3390/ijerph16183475

**Published:** 2019-09-18

**Authors:** Patryk Tarka, Agnieszka Chojecka, Olga Paduch, Aneta Nitsch-Osuch, Krzysztof Kanecki, Anna Kierzkowska

**Affiliations:** 1 Department of Social Medicine and Public Health, Medical University of Warsaw, ul. Oczki 3, 02-007 Warsaw, Poland; 2 Laboratory of Combating Infectious Agents and Biological Contaminations, Department of Bacteriology and Biocontamination Control, National Institute of Public Health—National Institute of Hygiene, ul. Chocimska 24, 00-791 Warsaw, Poland

**Keywords:** disinfection, EN 16615, ready-to-use alcohol-based wipes, contact time, bactericidal activity

## Abstract

Background: The effectiveness of ready-to-use disinfectant wipes was previously assessed in standardized suspension tests, which were inadequate because they ignored that the wipes are rubbed against a surface. Thus, we assessed the effectiveness of commercially available disinfectant wipes impregnated with an alcoholic solution according to the 16615 standard, which includes a test with mechanical action. Methods: According to the EN 16615 standard, under clean conditions, four squares (5 cm × 5 cm), placed next to one another, were marked on a test surface. *Enterococcus hirae*, *Pseudomonas aeruginosa,* and *Staphylococcus aureus* were inoculated on the leftmost square, and a wipe impregnated with an alcoholic solution was placed to the left of that square. Then, the wipe was pressed with a 2.5 kg weight and moved to the right and back to the left. After contact times of 1, 5, 10, or 15 min, we measured the reduction in bacterial load. Results: Alcohol-based ready-to-use commercial wipes did not show sufficient bactericidal activity at the contact times of 1, 5, 10 and 15 min. Wipes containing propan-1-ol and a mixture of propan-1-ol and propan-2-ol were active against *Pseudomonas aeruginosa* at the contact times of 1 min and 15 min. None of the examined wipes were active against *Enterococcus hirae* or *Staphylococcus aureus*. Conclusion: Bactericidal parameters of ready-to-use disinfectant wipes should be determined in surface tests, in addition to suspension tests, because suspension tests do not simulate the conditions under which disinfectant wipes are used in practice.

## 1. Introduction

The effectiveness of disinfection products that require mechanical action has not been tested adequately [1,2,3]. For example, suspension tests have been used to assess the effectiveness of solutions in which disinfectant wipes are impregnated, but they ignore that the wipes need to be rubbed against a surface [4,5]. Moreover, the fabric of disinfectant wipes may reduce the disinfectant’s activity. Although there are effectiveness tests for surface disinfection products (phase 2, step 2 test EN13697, prEN 17387), they apply large volumes of disinfectants (318 mL/m^2^), and they do not include the mechanical action of wiping.

In contrast, the EN 16615 European standard includes a standardized 4-field test that simulates the mechanical action of wiping a surface [6]. The test measures the reduction in contamination with bacteria and yeast that are inoculated on a flat polyvinyl chloride (PVC) surface [7]. The EN 16615 standard is useful particularly for disinfection products used in medical facilities [8]. Furthermore, in the EN 16615 standard, one can assess whether a given disinfectant wipe spreads microbial contamination over the test surface [7,9,10]. 

In medical facilities, disinfection wipes are typically applied to reduce microbial contamination on frequently used surfaces [3,11,12,13]. Therefore, disinfectants in which wipes are impregnated should have short contact times and a broad spectrum of anti-microbial action [2]. In practice, commercially available disinfection wipes are typically impregnated in alcohol-based solutions, and they are used to disinfect small surfaces [14,15]. 

This study assessed the bactericidal activity, according to EN 16615 standard, of several ready-to-use commercially available disinfection wipes impregnated with alcoholic solutions. 

## 2. Materials and Methods

### 2.1. Disinfection Wipes 

We used four types of commercially available disinfectant wipes (wipes A–D), with manufacturer-reported contact times of 30 s to 60 s (based on suspension tests, EN 13727; Table 1).

### 2.2. 4-Field Test

**Figure 1:**
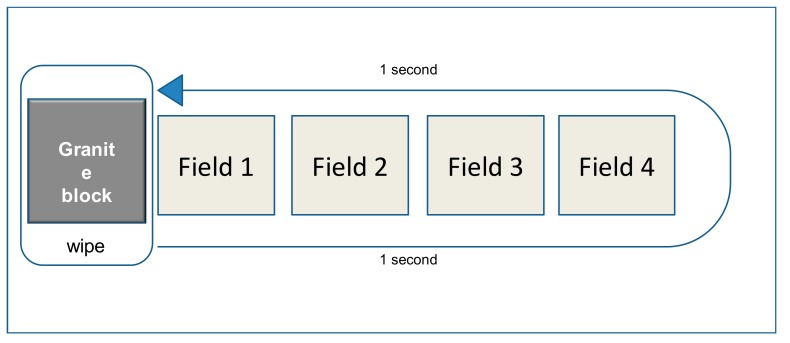
4-field test—description in text (methods).

According to the EN 16615 standard, we used the 4-field test to measure the effectiveness of disinfection wipes (Figure 1). Briefly, four square fields, 5 cm × 5 cm each, were marked on a flat PVC plate (20 × 50 cm^2^) layered with polyurethane (see Figure 1; DLW Solid Pur 521-029; Armstrong). Field 1 was inoculated with 50 µL of solutions that contained *Staphylococcus aureus* (ATCC 6538), *Pseudomonas aeruginosa* (ATCC 15442), and *Enterococcus hirae* (ATCC 10541) at concentrations of 1.5 × 10^9^ to 5.0 × 10^9^ colony forming units (CFU)/mL (bovine albumin solution, 0.3 g/L, clean conditions). The solutions were allowed to dry in a biosafety cabinet for about 1 h. Then, a disinfectant wipe was placed to the left of field 1, and it was pressed with a weight (granite block 12.1 cm × 8.6 cm × 8.6 cm, about 2.5 kg). The weight was moved to the right through all the fields with a constant movement that lasted 1 s, and then it was moved back again, over 1 s, to the original position. The wipes were weighed before and after the wiping. The difference in weight was taken as a measure of release of disinfectant solution from the wipes.

Immediately after wiping (N0) and at the end of contact time (1, 5, 10, 15 min), the bacteria were recovered from each test field with cotton stick swabs. For each test field (1–4), we used two swabs sticks and one tube with a neutralizer (30 g/L Polysorbate 80; 3 g/L lecithin; 1 g/L L-histidine; 30 g/L saponin in Tryptone Sodium Chloride diluent). First, a test field was rubbed with one swab stick soaked in the neutralizer, and the swab was washed in the neutralizer to prevent the action of disinfectant. This procedure was repeated once, and the tip of the swab stick was cut off and left in the neutralizer tube. Next, the test field was rubbed with a dry swab, which was also placed in the neutralizer tube, and the tube was shaken. After a neutralization period (15 s for the contact time <15 min; 5 min for the contact time ≥15 min), 1 mL samples of neutralized test suspensions were incubated on agar plates in quadruplicate at 37 °C for 48 h. The bacterial load was counted and expressed in lg CFU/mL. 

### 2.3. Controls for the 4-Field Test 

We used the following controls in the 4-field test: (1) Initial bacterial load (N)—density of bacteria incubated from the test suspension; (2) drying control (Dc0)—density of bacteria recovered from a dried up inoculate from a separate plate (to assess the loss of bacteria during drying); (3) contact time control (Dct )—density of bacteria recovered from a separate plate after a given contact time (reference for calculation of bacterial load reduction); (4) water control—bacteria recovered from fields 1–4 that were wiped with standard wipes (SCA Tork) soaked for 30 min in 16 mL of sterile distilled water with the addition of 0.1% Polysorbate 80; (5) neutralizer control—no toxicity of the neutralizer against the tested bacteria was confirmed in the neutralizer control procedure.

According to the EN 16615, effective bactericidal activity was defined as ≥5 lg reduction for *E. hirae, P. aeruginosa,* and *S. aureus* on test field 1 and bacterial load of ≤50 CFU per 25 cm^2^ on test fields 2 to 4 for each test organism. Bacterial spread with wiping was confirmed when bacterial load was >50 CFU per 25 cm^2^ on test fields 2 to 4. 

### 2.4. Statistical Analyses 

Data were analyzed with Microsoft Excel 2007. The mean values ± standard deviations are presented. 

## 3. Results

### 3.1. Release of Disinfectant Solution from Wipes 

The control water wipes released 1.33 ± 0.2 g of water. The amount of disinfectant released from wipes A was 1.26 ± 0.6 g, from wipes B was 0.75 ± 0.1 g, from wipes C was 0.87 ± 0.2 g, and from wipes D was 0.62 ± 0.1 g. 

### 3.2. Recovery of Bacteria from Test Fields 

**Figure 2:**
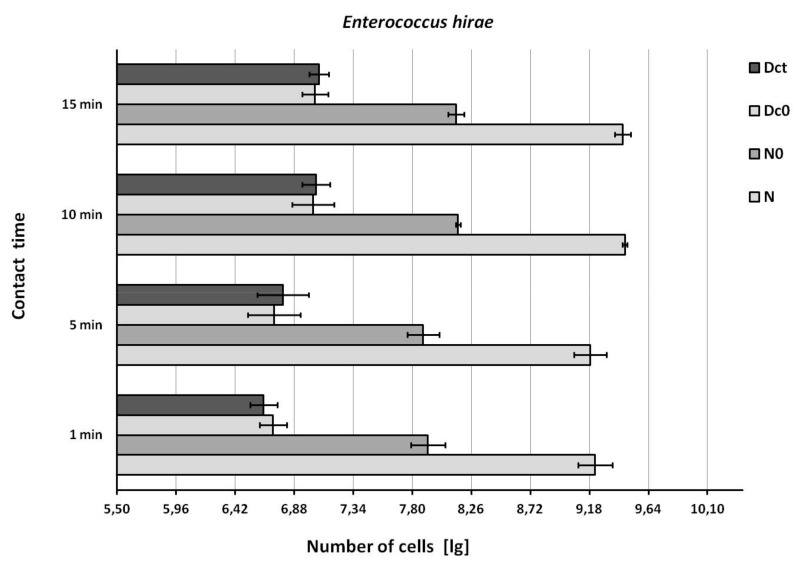
Recovery of *Enterococcus hirae* from the surface. N = density of test suspension (basic limit: 9.17 ≤ lg, N ≤ 9.70); N0 = density of the test suspension at the beginning of the contact time 0 (basic limit: 7.88 ≤lg, N0 ≤ 8.40 ); Dc0 = drying control at the contact time 0 (basic limit: 6.88 ≤ lg, Dc0 ≤ 8.40); Dct = drying control at the end contact time t (basic limit: 6.88 ≤ lg, Dct ≤ 8.40). Shown are means and standard deviations.

**Figure 3:**
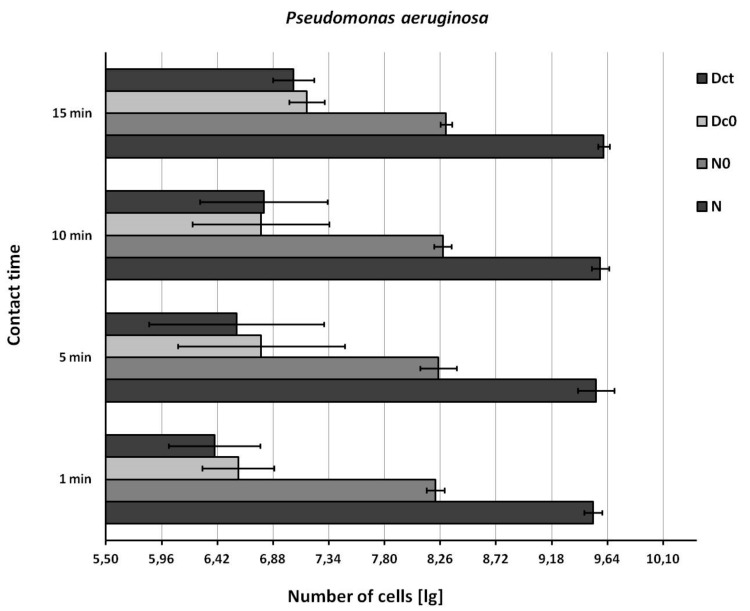
Recovery of *Pseudomonas aeruginosa* from the surface. N = density of test suspension (basic limit: 9.17 ≤ lg, N ≤ 9.70); N0 = density of the test suspension at the beginning of the contact time 0 (basic limit: 7.88 ≤lg, N0 ≤ 8.40 ); Dc0 = drying control at the contact time 0 (basic limit: 6.88 ≤ lg, Dc0 ≤ 8.40); Dct = drying control at the end contact time t (basic limit: 6.88 ≤ lg Dct ≤ 8.40). Shown are means and standard deviations.

**Figure 4:**
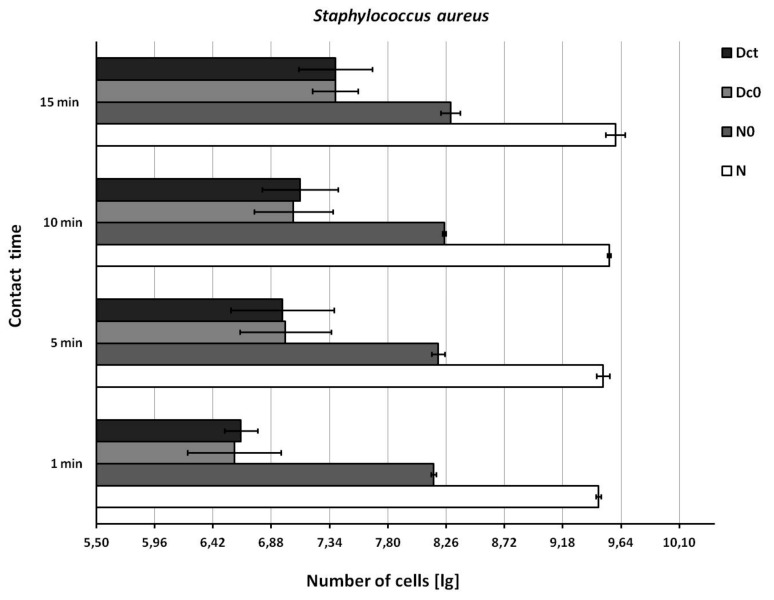
Recovery of *Staphylococcus aureus* from the surface. N = density of test suspension (basic limit: 9.17 ≤ lg, N ≤ 9.70); N0 = density of the test suspension at the beginning of the contact time 0 (basic limit: 7.88 ≤ lg, N0 ≤ 8.40 ); Dc0 = drying control at the contact time 0 (basic limit: 6.88 ≤ lg, Dc0 ≤ 8.40); Dct = drying control at the end contact time t (basic limit: 6.88 ≤ lg, Dct ≤ 8.40). Shown are means and standard deviations.

The recovery of bacteria from the test suspension (N) and from the test surface immediately after wiping (N0) was in accordance with the EN 16615 standard (Figure 2, Figure 3 and Figure 4). 

The recovery of bacteria from the drying control (Dc0) and contact time control (Dct) was similar, but below the EN 16615 standard (6.88 ≤ lg ≤ 8.40, Figure 2, Figure 3 and Figure 4) when the test bacterial suspension was in the lower range. The recovery of bacteria from the drying control (Dc0) and contact time control (Dct) improved to acceptable values when the concentration of the test suspension was increased (*E. hirae,* 9.46 lg; *P. aeruginosa*, 9.61 lg; *S. aureus*, 9.50 lg). 

### 3.3. Reduction of Bacterial Load after Wiping

The reduction in bacterial load on field 1 after wiping did not meet the benchmark of the EN 16615 standard. None of the wipes (A–D) reduced bacterial loads by >5 lg for all bacterial strains (Table 2). Reduction of bacterial load >5 lg was observed for wipes B only in the test with *P. aeruginosa* (Table 2). The average bactericidal activity of the tested wipes ranged from 2.76 lg ± 0.73 at 1 min of contact time to 3.50 lg ± 0.56 at 10 min of contact time. The mean bactericidal activity against Gram-positive bacteria was 2.47 lg ± 0.14 to 3.69 lg ± 1.17, whereas that against Gram-negative bacteria was 2.89 lg ± 1.49 to 4.35 lg ± 0.76.

### 3.4. Spreading of Bacteria after Wiping

Some wipes spread bacteria from field 1 to fields 2–4 (> 50 CFU/25 cm^2^, Table 2), although the bacterial spread was reduced compared to the water control (Table 3). There was no pattern between the duration of contact time and bacterial spread (Table 2). 

## 4. Discussion 

In this study, the effectiveness of disinfectant wipes impregnated with alcohol-based solutions did not meet the benchmark of the EN 16615 standard, which requires a test with mechanical action. Moreover, wiping spread bacteria in some experiments. 

The EN 16615 standard, unlike suspension standards, simulates many practical aspects of disinfection with wiping in medical facilities including the surface type, test microorganisms that adhere to the surface, and the mechanical action of wiping [2,7]. Moreover, the recovery of microorganisms from the surface and microbial spread during wiping is also evaluated [7]. Thus, the EN 16615 standard is more demanding than the suspension standards. 

In medical facilities, disinfectant wipes are typically applied for quick disinfection of small and frequently used surfaces. Disinfection wipes are often impregnated with alcohol, because alcohol kills bacteria, yeast, fungi, and viruses after short contact times. As a result of their low molecular weight, alcohol evaporates readily from surfaces without a residual effect [16,17]. However, the volatility of alcohol can decrease contact times and thus also reduce their antimicrobial activity [17]. 

In the 4-field test, the disinfectant released from a wipe is spread as a thin layer, which evaporates rapidly from the surface at room temperature. Short contact times of the disinfectant with microorganisms may worsen the effectiveness of the wipes [18]. In our study, the amount of disinfectant released from particular wipe types differed, but this difference did not have any effect on the bactericidal activity. Gebel et al. showed that small volumes of disinfectant may not cover a given surface and thus have insufficient antimicrobial activity [18]. Our research supports this view, because we found that extending contact times, beyond those put forward in the EN 16165 standard (<5 min), did not increase bactericidal activity of the alcohol-impregnated wipes, possibly because low volumes of alcohol evaporate quickly 

In our study, the alcohol content in the disinfectant wipes was 50%–90% [16,17]. Despite these differences, all the wipes had similar bactericidal activity, which did not meet the EN 16615 benchmark. Wipes C, containing propan-1-ol and propan-2-ol, had the highest bactericidal activity (against *P. aeruginosa*), which is consistent with earlier reports [19,20]. Similar to previous studies, we found that Gram-negative bacteria were more susceptible to alcohol than were Gram-positive bacteria [17,19]. Andersen et al. showed that the bacterial reduction with ethanol wipes did not meet the EN 16615 benchmark (< 3 lg) [21]. Our results are in line with that report. 

Disinfectant wipes should prevent the spread of bacteria on surfaces [10]. Compared to water control, the wipes investigated in our study reduced bacterial spread. However, we observed bacterial spread, as defined by the EN 16615 standard, in some experiments (Table 2). Other studies showed that the spread of microorganisms may depend on the type of wipe, impregnating substance (disinfectant or detergent), wiping technique, and the type of microorganism [2,13,22]. The bactericidal activity of disinfectant wipes may be improved by selecting the most effective alcohol or adding substances that reduce surface tension, to provide better surface hydration, or emollients, in order to prevent the rapid evaporation of alcohol [23]. Moreover, larger disinfectant volumes for impregnating wipes may increase their bactericidal activity [18]. 

Our study had limitations. The EN 16615 standard requires a 4-field test with yeasts. However, ineffective action against bacteria means that a given wipe type fails the EN 16615 standard. Thus, we did not do experiments with yeasts. We did not compare the EN 16615 standard with other standards that can be valid for disinfection wipes, such as the ASTM E2967-15 standard. 

## 5. Conclusions

Commercially available disinfectant wipes impregnated with alcohol may not meet the EN 16615 standard. The contact times reported by manufacturers may be too short to enable effective bactericidal action of wipes. The contact times are evaluated in tests with larger volumes of disinfectants, and thus without mechanical action (EN13727) are not valid for disinfectant wipes. 

Future studies should compare the EN 16165 standard with the American standards ASTM E2967-15 for disinfectant wipes. 

## Figures and Tables

**Table 1:** Formulations of the ready-to-use commercially available wipes according to the manufacturers.

Wipes	Formulations of Ready-to-Use Commercial Wipes in 100 g Solution	Test Method	Organic Load	Contact Time
A	25 g ethyl alcohol, 35 g propan-1-ol	No information	No information on the label*	1 min
B	50 g propan-1-ol, 0.075 g didecyldimethylammonium chloride	No information	No information on the label *	1 min
C	35 g propan-2-ol, 25 g propan-1-ol	EN 13727	Clean condition 0.3 g/L bovine albumin solution Dirty condition 3 g/L bovine albumin solution + 3 mL/l sheep erythrocytes	1 min
D	45 g ethyl alcohol, 30 g propan-2-ol, 0.5 g didecyldimethylammonium chloride	No information	No information on the label*	30 s

**Table 2:** Log-reduction (lgR) and the spread of bacteria (Eh = *E. hirae*, Ps = *P. aeruginosa* and Sa = *S. aureus*) on the test fields from T2 to T4 per 25 cm^2^ after wiping with the tested wipes A, B, C, D at the contact times from 1 to 15 min. Values indicating bacterial spread (>50 CFU/25 cm^2^) are shown in bold.

Wipes/Test Organisms	Contact Times							
	1 Min		5 Min		10 Min		15 Min	
	lgR	T2-T4	lgR	T2-T4	lgR	T2-T4	lgR	T2-T4
**A/Eh**	2.48	**800**	4.26	3	2.78	0	2.84	5
**A/Ps**	2.69	2	1.32	7	4.20	0	2.90	**460**
**A/Sa**	2.36	3	2.65	0	2.72	0	2.74	0
**B/Eh**	2.60	**550**	4.60	0	4.18	**146**	2.85	8
**B/Ps**	5.05	28	5.37	0	5.37	0	5,39	0
**B/Sa**	3.87	1	3.78	0	3,44	44	3.32	5
**C/Eh**	2.82	2	3.08	1	2.96	2	2.77	38
**C/Ps**	1.78	0	2.57	2	4.30	3	5.25	3
**C/Sa**	3.06	**138**	3.06	**138**	2.59	3	3.30	5
**D/Eh**	2.52	**447**	2.39	13	2.88	7	2.96	0
**D/Ps**	2.04	**92**	2.56	0	3.54	0	2.60	35
**D/Sa**	2.35	**125**	2.67	**108**	2.99	17	3.32	0

**Table 3:** The mean number of recovered bacteria (Eh = *E. hirae*, Ps = *P. aeruginosa*; Sa = *S. aureus*) on the test fields from T2 to T4 after wiping with water (Nw).

Water Control (Nw)/ Test Organisms	Contact Times			
	1 Min	5 Min	10 Min	15 Min
	T2-T4 cfu/25 cm^2^	T2-T4 cfu/25 cm^2^	T2-T4 cfu/25 cm^2^	T2-T4 cfu/25 cm^2^
**Nw/Eh**	16,500	16,500	2947	2660
**Nw/Ps**	2121	277	3667	1216
**Nw/Sa**	9490	11,133	4204	13,750
